# LONGITUDINAL SEX DIFFERENCES IN FRAILTY AND AGING IN UM-HET3 MICE

**DOI:** 10.1093/geroni/igae098.2767

**Published:** 2024-12-31

**Authors:** Judy Wu, Brady Samuelson, Elizabeth Betz, Alice Kane

**Affiliations:** Institute for Systems Biology, Seattle, Washington, United States; Institute for Systems Biology, Seattle, Washington, United States; Institute for Systems Biology, Washington, United States; Institute for Systems Biology, Seattle, Washington, United States

## Abstract

Frailty is a common geriatric syndrome characterized by age-associated decline and vulnerability to adverse health outcomes. Frailty can be quantified using frailty indices (FIs) which count the number of health deficits in an individual. Sex differences are observed in aging with women living longer than men, despite women being frailer. Our goal is to assess sex differences in behavioral and molecular assessments of aging, and how frailty may influence these changes. Across the lifespan of UM-HET3 male and female mice(n=37) we are longitudinally measuring FI score, behavioral assessments and blood outcomes including white blood cell counts (WBC) and glucose levels. Preliminary data from the first 3 timepoints unexpectedly suggest frailty scores decrease over time(p=9.98e-13), with no clear sex differences. For nesting, females have higher scores at all timepoints (p=7.71e-04). Males have higher WBC than females (p = 1.29e-06), with no change over time. Glucose levels increase overtime for both sexes, (p=2e16), but males have lower glucose counts than females (p=5.25e-3). Frailty is associated with lower glucose levels in both sexes (p= 4.45e-05). Heart rate is higher in females (p= 3.52e-04) and increases over time for both sexes (p=3.59e-03). Additionally, higher frailty scores are associated with lower heart rates(p=0.0236) in both sexes. Data collection is ongoing and we expect to see more significant effects of sex, age, and frailty as the mice grow older. This work will help understand the impact of frailty and sex differences on aging.

